# Analysis of zinc and copper levels in very low birth weight infants using human milk additives: phase 1 trial findings

**DOI:** 10.1016/j.jped.2024.08.007

**Published:** 2024-10-05

**Authors:** Renata G. Oliveira, Maria C. Achcar-Feih, Vicky Nogueira-Pileggi, Adriana Carnevale-Silva, Fabio Carmona, Davi C. Aragon, Mariana M. Oliveira, Luciana M.M. Fonseca, Larissa G. Alves, Vanessa S. Bomfim, Tânia M.B. Trevilato, Isabela Spido-Dias, Fabio V. Ued, Marisa M. Mussi-Pinhata, Jose S. Camelo

**Affiliations:** a Department of Pediatrics, Ribeirão Preto Medical School, University of São Paulo, Brazil; b Department of Maternal-Infantile and Public Health, Nursing School of Ribeirão Preto, University of São Paulo, Brazil; c Human Milk Bank, Clinics Hospital, Ribeirão Preto Medical School, University of São Paulo, Brazil; d Laboratory of Pediatrics, Division of Metals and Rare Diseases, Clinics Hospital, Ribeirão Preto Medical School, University of São Paulo, Brazil; e Department of Health Sciences, Ribeirão Preto Medical School, University of São Paulo, Brazil

**Keywords:** Zinc, Copper, Human Milk, Premature Infants, Newborn

## Abstract

**Objective:**

The aim of this study was to assess whether the micronutrients zinc and copper, provided by human milk additives, are sufficient for very low birth weight preterm infants.

**Method:**

A phase 1 randomized double-blind controlled trial was conducted with very low birth weight preterm infants. This is a secondary analysis of copper and zinc. Sixty-six newborns were part of the initial sample, with forty participating and reaching the final stage of the study. Inclusion criteria were: gestational age less than 37 weeks, birth weight greater than or equal to 750 g and less than or equal to 1500 g, small or appropriate for gestational age, exclusively receiving human milk at a volume greater than or equal to 100 mL per kilogram per day, and hemodynamically stable. Participants were randomly assigned to two groups: intervention, Lioneo (received human milk with additive based on lyophilized human milk), *n* = 20, and control, HMCA (received human milk with commercial additive based on cow's milk protein), *n* = 20, and their serum levels of zinc and copper were measured on the first and twenty-first days.

**Results:**

There was a reduction in intragroup zinc serum levels from the first to the twenty-first day of the study (*p* < 0.01). There was no intergroup difference. No difference was found in serum copper levels.

**Conclusion:**

Human milk additives were not sufficient to maintain adequate zinc serum levels in very low birth weight newborns. It was not possible to affirm whether human milk additives were sufficient to maintain adequate serum copper levels in the studied sample. UTN: U1111–1220–0550.

## Introduction

According to the World Health Organization, an average of 15 million premature babies are born annually worldwide.^1^ Newborns with very low birth weight (VLBW, < 1500 g) have difficulty in meeting their nutritional needs, often requiring additional supplements to human milk to enhance calorie, protein, and micronutrient provisions, such as calcium, phosphorus, zinc, and copper.^2^^,^^3^

The composition of human milk has been studied countlessly because of its complexity.^4^^,^^5^ Human milk comprises carbohydrates, lipids, proteins, vitamins, minerals, growth factors, and oligosaccharides. Variations occur depending on the gestational age, time elapsed after birth, maternal diet, interval between feeding, and milk storage time. Even during breastfeeding, there may be variations in the energy composition of milk depending on the time it is stored in the lactiferous ducts.^6^ Zinc, with its high bioavailability, is essential for several enzymatic processes, including cell growth and multiplication, cellular membrane formation, and metabolism of carbohydrates, lipids, proteins, and nucleic acids.^7^^,^^8^ In premature newborns, serum zinc levels decline after birth, reaching their lowest point at 6–12 weeks of age,^9^ which makes them more susceptible to zinc deficiency and contributes to osteopenia of prematurity, growth retardation, bronchopulmonary dysplasia, and digestive difficulties.^10^

Copper is involved in numerous enzymes and influences iron metabolism.^11^ Approximately 75 % of fetal copper accumulation occurs during the last trimester of pregnancy, rendering premature newborns more vulnerable to copper deficiency, which can lead to growth difficulties, hypotonia, apnea, hypothermia, alterations in bone metabolism, anemia, and increased susceptibility to infections owing to altered neutrophil function.^7^

Despite the extremely detrimental effects of copper and zinc deficiencies in premature newborns, serum levels of these micronutrients are seldom assessed in healthcare settings because of the lack of inclusion in routine procedures, the absence of protocols guiding this practice, and a limited number of studies confirming this necessity.^12^ Therefore, the authors hypothesized that human milk fortifiers alone may not adequately meet the zinc and copper requirements of newborns with VLBW. This study aimed to assess whether zinc and copper, provided by human milk additives, are sufficient for premature newborns with VLBW.

## Material and methods

This study was a secondary analysis focusing on copper and zinc assessed through serum levels in premature newborns over a specified period. The original study involved developing a lyophilized human milk product as an additive in human milk to provide optimal nutrition for newborns with VLBW. Additionally, it includes the analysis of a phase 1 randomized, controlled, double-blind clinical trial involving premature newborns with VLBW (< 1500 g) admitted to the neonatal intensive and intermediate care units between May 2019 and April 2020. Currently, the study is concluding in phase 2, evaluating efficacy using the same design.

This study was performed at the Neonatal Intensive and Intermediate Care Center of the Children's Hospital, Ribeirão Preto Medical School, University of São Paulo. The study participants were premature newborns with VLBW meeting the inclusion criteria; gestational age at birth < 37 weeks, birth weight 750–1500 g, small or appropriate for gestational age, exclusively receiving human milk at ≥ 100 mL/kg/day, hemodynamically stable (without vasoactive drugs, blood pressure within the normal range and/or good peripheral perfusion, capillary refill time < 3 s, palpable peripheral pulses, and oxygen saturation > 90 %), and whose parents or guardians signed the informed consent form. Only one identical twin participated in this study. Fraternal twins were included. The exclusion criteria were as follows; newborn large for gestational age, presence of major congenital malformations, such as congenital heart defects, and periventricular or intraventricular hemorrhage grades III and IV.

The milk was sourced from breastfeeding mothers who donated surplus milk without compromising the feeding of their own children. The donors received instructions according to the regulations of the Human Milk Bank Network in Brazil^13^ and underwent clinical and serological screening. Human milk supplemented with lyophilizate (Lioneo) or commercial additive FM85®, Nestlé® (HMCA), was prepared based on a previous description of the preclinical and clinical phases, and the concentrated human milk was considered physically and chemically stable, microbiologically safe, and safe and tolerable for premature newborns with VLBW after phase 1.^14^ Zinc and copper were analyzed by atomic absorption spectrophotometer Varian® Australia Pty Ltd (Mulgrave, Victoria, Australia) immediately after thawing the samples in a water bath at 37 °C using the ultrasonic method for sample homogenization via the Sonicator-MIRIS® (Uppsala, Sweden).

The participants were divided into two groups by electronic randomization via a computer-generated list and simple randomization at a 1:1 ratio. Allocation concealment was ensured using Research Electronic Data Captures (REDCap). The minimum sample size required by the National Institute of Health/Food and Drug Administration (NIH/FDA) was 20 patients, and this phase 1 study included 20 individuals in each group. Children are considered a vulnerable population, and according to the FDA recommendations, phase 1 studies involving normal or stable individuals should include the smallest possible number of patients to minimize exposure to potential adverse events and ensure safety and tolerability. Most phase 1 studies typically involve 20–80 participants (Good Review Practice: Clinical Review of Investigational New Drug Applications, 2013; available at chrome-extension://efaidnbmnnnibpcajpcglclefindmkaj/https://www.fda.gov/media/87621/download).

Parents or guardians, assisting teams (physicians, nurses, and other hospital staff), and researchers were blinded. Only the three nutritionists responsible for distributing milk into syringes were not blinded. The patients were assessed daily by a research nurse, and data were entered into the REDCap database through form completion. Data included body weight, abdominal circumference, volume of milk effectively ingested, and major events that occurred. All data were cross-checked by the project manager and any inconsistencies were resolved at the end of each week. The patients underwent laboratory tests, including measurement of serum zinc and copper levels, on days 1 and 21.

The intervention group (group A) received Lioneo (75 mL of human milk with 7 g of lyophilized human milk), while the control group (group B) received HMCA, human milk with an additive based on cow's milk protein (FM85®, Nestlé®), at a concentration of 4 %. Both groups were followed in similar manners. The three non-blinded nutritionists were responsible for preparing diets according to their daily medical prescriptions. The diets for both groups had a similar appearance and smell, making them indistinguishable at the bedside, and were sent encoded to the neonatal units, with no possibility of identification. All newborns received 100–160 mL/kg/day of milk and were followed for 21 consecutive days. The medical team could decide to interrupt the diet depending on the neonatal unit protocol. The project team did not interfere with the medical decisions. Breast milk intake from the mothers was prioritized and encouraged.

Detailed data analysis is reported in tables containing measures of central tendency, dispersion, absolute frequencies, and relative frequencies. The means of the groups and time points were compared using a linear mixed-effects model. Multiple comparisons were conducted by estimating the orthogonal contrasts. The software used was SAS® 9.4.

All precepts of Resolution 466/2012 of the National Health Council, which regulates research involving humans, are respected. This study was approved by the Research Ethics Committee (CAAE: 96,682,318.2.0000.5440) and registered in the Brazilian Clinical Trials Registry (REBEC) with the Universal Trial Number (UTN): U1111-1220-0550, available at http://www.ensaiosclinicos.gov.br/rg/RBR-8nnpfm/. Registration on the Brazilian Platform: 96,682,318.2.0000.5440. An informed consent form was signed by the parents or guardians of the participants.

## Results

### Preclinical phase

Fifty breastfeeding mothers participated in the preclinical study, with a mean age of 30.45 years, mean weight of 67.5 kg, mean height of 1.63 m, mean BMI of 25.2 kg/m², and mean gestational age of 38.43 weeks.^14^ After undergoing the described procedures, lyophilized human milk exhibited an average Dornic acidity level of 4.34° D. Protein, carbohydrate, lipid, zinc, copper, and osmolality was measured as previously described.^15^ The zinc and copper levels in concentrated human milk with lyophilizate were 203.89 ± 126.2 μg/100 mL and 48.30 ± 19.99 μg/100 mL, respectively.

### Clinical phase

The sample's pathway can be seen in the flowchart in the Supplementary Material. Sixty-six patients were screened, and 40 neonates were included between May 6, 2019, and April 6, 2020. Data from all the participants were collected and entered into REDCap. The 40 newborns were randomly assigned to two groups; the intervention group, Lioneo (received human milk with an additive based on lyophilizate), *n* = 20, and the control group, HMCA (received human milk with an additive based on cow's milk protein, FM85®), *n* = 20. There were 11 girls in the Lioneo group (52.38 %) and 10 girls in the HMCA group (50 %). The participant characteristics in both groups on days 1 and 21 are presented in Table 1.^16^

**Table 1:** Characterization of preterm newborns in the intervention (Lioneo) and control (FM85®) groups from the phase 1 clinical trial.

	Lioneo group (A)		**HMCA** group (B)	
	Mean	Standard deviation	Mean	Standard deviation
Birth weight (g)	1220,00	200,69	1219,30	204,78
Gestational age (weeks)	30,45	2,68	29,70	1,75
Length at birth (cm)	38,08	2,17	37,77	2,89
Head circumference at birth (cm)	27,50	1,67	27,03	1,41
Inclusion age (days)	11,40	8,92	12,05	6,44
Weight at the first day of study (g)	1222,80	178,09	1254,30	200,43
Length at first day of study (cm)	38,46	2,33	38,85	2,20
Head circumference at the first day of study (cm)	26,34	2,13	26,27	1,99
Weight at 21st day of study (g)	1565,30	263,79	1678,50	262,60
Length at 21st day of study (cm)	41,14	1,92	42,05	2,14
Head circumference at 21st day of study (cm)	28,54	2,29	28,39	2,07

g, grams; cm, centimeters; Lioneo (A) group received human milk with an additive based on lyophilized human milk. HMCA (B) group received human milk with an additive based on hydrolyzed cow's milk protein.

Serum zinc and copper levels were measured in participants from both groups on days 1 and 21. A detailed description of the serum zinc and copper levels is shown in Table 2. There was a reduction in serum zinc levels on day 21 compared to that on day 1 in both groups. Statistical analysis was conducted to determine whether there was a statistically significant difference in the means of serum zinc and copper levels in the intervention and control groups, as shown in Table 3.

**Table 2:** Measures of central tendency and dispersion of serum copper and zinc levels in μg/100 mL, for the intervention and control groups, on days 1 and 21 of the clinical trial.

Group	Day of the study	n	Variable	Mean	SD	Min	1st Quartile	Median	3rd Quartile	Max
	1	20	Copper	56,7	17,4	32,8	40,8	56,1	62,8	97,2
**Lioneo**		20	Zinc	81,7	24,5	26,5	74,5	84,8	90,3	135,0
	21	20	Copper	48,9	16,4	25,2	36,0	43,0	58,0	91,0
		20	Zinc	66,7	18,9	40,5	49,3	65,0	80,0	113,0
	1	20	Copper	61,3	26,0	21,9	45,4	53,0	72,3	130,6
**HMCA**		20	Zinc	85,0	16,4	54,5	71,8	87,3	92,3	115,0
	21	20	Copper	55,7	17,1	31,8	42,0	55,5	69,0	90,4
		20	Zinc	60,9	10,8	44,0	51,0	63,8	67,3	83,5

n, number of participants; SD: standard deviation; Min: minimum; Max: maximum; Lioneo group: newborns who received human milk supplemented with a human milk-based additive; FM85® group: newborns who received human milk supplemented with hydrolyzed cow milk protein-based additive.

**Table 3:** Comparisons by mean difference of serum copper and zinc levels, for the intervention and control groups, on days 1 and 21 of the clinical trial.

Variable	Comparisons	Mean difference	p-value	CI 95 % LL	CI 95 % UL
Copper	A – B (D1)	−45,6	0,47	−171.4	80,1
	A – B (D21)	−67,7	0,28	−193.5	58,1
	D1 - D21 (A)	78,0	0,11	−19.1	175,1
	D1 - D21 (B)	55,9	0,25	−41.2	153,1
Zinc	A – B (D1)	−33,5	0,57	−150.8	83,8
	A – B (D21)	58,2	0,32	−59.1	175,6
	D1 - D21 (A)	149,5	**<0,01**	67.0	231,9
	D1 - D21 (B)	241,2	**<0,01**	158.7	323,7

D1, first day of the study; D2, twenty-first day of the study; Lioneo group (A), newborns who received human milk with a human milk-based additive; HMCA group (B), newborns who received human milk with a cow milk protein-based additive; 95 % CI, 95 % confidence interval; LL, lower limit; UL, upper limit.

**Figure 1:**
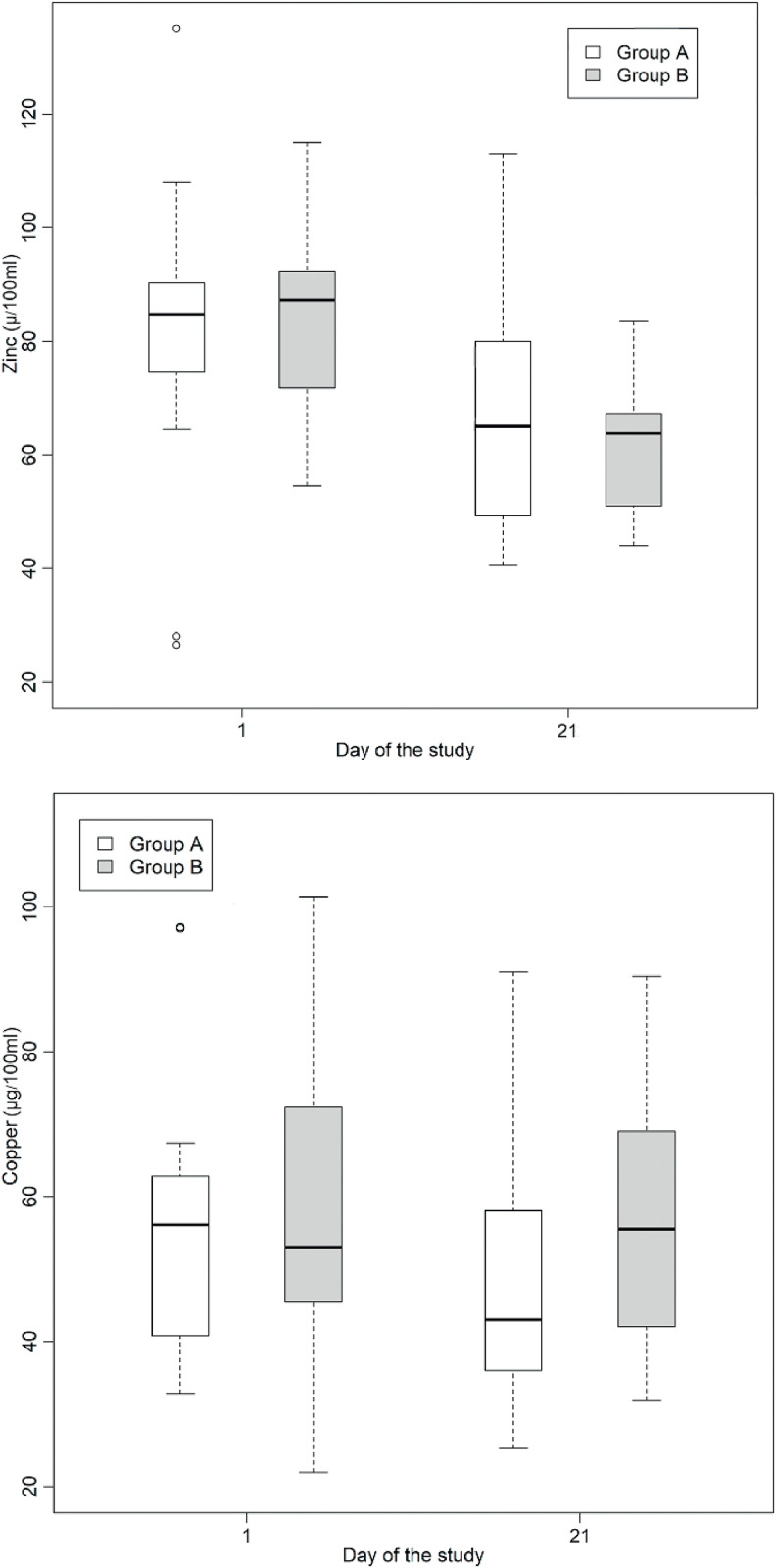
Boxplots illustrating the fluctuations in serum (µg/dL) zinc and copper levels among preterm newborns with very low birth weight who were administered either a human milk-based additive (A) or cow milk protein-based additive (B) during the phase 1. Statistical analysis reveals a significant difference in zinc levels between days 1 and 21 within both cohorts (*p* < 0.05).

Serum zinc levels decreased in both groups (Table 3 and Figure 1) with no statistically significant difference between the Lioneo and HMCA groups. Nevertheless, in comparing the serum zinc levels of days 1 and 21, there was a statistically significant difference in the control and Lioneo groups. No differences were found in comparing the copper levels in the intervention and control groups, nor in that of the serum levels of days 1 and 21 within the same group.

## Discussion

The present phase 1 clinical trial demonstrated that human milk additives, based on lyophilized human milk or hydrolyzed cow milk protein, were insufficient to maintain adequate serum zinc levels in premature newborns with VLBW. The data indicated an intragroup reduction between days 1 and 21, with no intergroup differences. Regarding copper, no difference was found between or within groups; therefore, it was impossible to ascertain whether the additives were sufficient to maintain adequate serum copper levels.

Maternal nutritional status affects the levels of macro- and micronutrients in newborns; however, uncertainties regarding the necessity of gestational supplementation of zinc and copper remain. A clinical trial conducted at the Federal University of Mato Grosso do Sul in 2009 assessed maternal zinc supplementation and serum zinc and copper levels in premature newborns using control and intervention groups that received supplementation. There were no differences in zinc levels in breast milk, serum levels in newborns, and anthropometric measurements between both groups; however, there was a decrease in serum copper levels in mothers receiving zinc supplementation.^17^

The Brazilian Society of Pediatrics references in its 2012 outpatient follow-up manual for high-risk preterm infants zinc supplementation for preterm newborns, initiated at 36 weeks corrected gestational age until completion of six months corrected age. However, it remains not routine in many healthcare facilities in Brazil owing to financial constraints and studies are relatively new and present conflicting results, which are being increasingly clarified.^18^ Additionally, the lack of experience among teams in managing and controlling this supplementation delays or renders this practice unfeasible. A systematic review conducted by Cochrane in 2021, which included five large studies on zinc supplementation in preterm infants, comprising 482 preterm infants, concluded that zinc supplementation reduced mortality but had little-to-no effect on comorbidities, such as bronchopulmonary dysplasia, retinopathy of prematurity, bacterial sepsis, and necrotizing enterocolitis. Weight gain and linear growth improved, with little-to-no effect on head circumference.^19^ Highlighting conflicting results, a prospective American cohort study conducted in 2019 at a tertiary hospital with 105 preterm infants, in which enteral zinc supplementation was administered during the first two weeks of life, found a positive association between weight gain and head circumference growth, both statistically significant (*p* < 0.01).^20^ A Japanese retrospective study showed that infants born at < 30 weeks of gestation had a 46 % reduction in serum zinc levels at one month of age and all infants born at < 24 weeks of gestation had zinc deficiency at two months of age.^21^

Studies on copper are scarce and uncertain. A cohort study conducted in Spain in 2016 analyzed serum copper levels in 596 newborns, seeking correlations with days of life, feeding type, inflammatory status, and prematurity, with prematurity as the most important factor correlated with low serum copper and ceruloplasmin levels.^22^ Another case-control study conducted in India with 256 preterm infants found an association between retinopathy of prematurity, low maternal serum copper and zinc levels, and low levels of zinc and vitamin A in the umbilical cord blood.^23^ A retrospective cohort study conducted in Japan in 2017 involving premature newborns and data collected from medical records found that serum copper levels in the umbilical cord blood were significantly lower than the maternal serum levels, suggesting that copper does not diffuse through the placenta and requires active transport to reach the fetus. However, the study was observational and the sample size was small.^24^ A case-control study evaluated maternal serum zinc and copper levels with preterm and term deliveries and concluded that serum copper levels were lower in mothers with premature delivery.^25^

Studies of zinc and copper in human milk are scarce. The Brazilian scientific literature lacks studies on micronutrients in human milk and their implications for premature newborns owing to high ethical and methodological rigor, including appropriate design, sufficient sample size, and minimal bias, for the results to have external validity. Copper and zinc deficiencies in premature newborns contribute to increased morbidity and mortality due to a higher predisposition to anemia, vulnerability to infections and bronchopulmonary dysplasia, and deficits in growth, digestive difficulties, osteopenia of prematurity, and neuropsychomotor developmental delay, making this topic increasingly relevant.^7^^,^^9^

The present study revealed a significant decrease in serum zinc levels for 21 days in preterm newborns with VLBW. A similar trend may occur with copper; however, the sample size was a limitation because this was a phase 1 study. Therefore, cow milk protein and human milk-based additives were insufficient to maintain adequate serum zinc levels in premature newborns with VLBW. Further studies are needed to assess the adequacy of copper and zinc in preterm newborns with VLBW. The authors speculate that zinc supplementation commences from the maximum volume of human milk supplemented with human or commercial additives, which should be confirmed with a larger sample size in phase 2 studies.

## Declaration of generative AI and AI-assisted technologies in the writing process

The authors used ChatGPT3.5 (chat.openai.com) in preparing this work only to improve the readability. After using this tool/service, the authors reviewed and edited the content as needed and are responsible for the content of the publication.

## Financial support

This study was supported by the 10.13039/501100003593Conselho Nacional de Desenvolvimento Científico e Tecnológico (CNPq), Grant #: 421721/2017-0 to JSCJr. and Bill and Melinda Gates Foundation, Grant # OPP1107597 to JSCJr. The funders had no role in the design and results of this study.

## Conflicts of interest

The authors declare no conflicts of interest.

## Appendix Supplementary materials

Image, application 1
