# Supplementary material for: Analysis of zinc and copper levels in very low birth weight infants using human milk additives: phase 1 trial findings
Source: J Pediatr (Rio J). 2024 Oct 5;101(2):187–93. doi: 10.1016/j.jped.2024.08.007 (PMC11889681; doi:10.1016/j.jped.2024.08.007)
Supplement: Supplementary file 1 [file mmc1.docx]

**JPED-D-24-00212 – Supplementary Material**

**Selection**

Elegible patients

(n = 66)

Excluded (n = 26):

- Criteria for inclusion were not met (n=9)

- Withdrawal (n=10)

- Other reasons (n=7)

**Randomization**

(n=40)

Allocated to the control group,

FM85^®^ Group

(n=20)

Allocated to the intervention group, Lioneo Group (n=20)

**Alocation**

Loss to follow-up

(n =0)

**Follow-up**

Loss to follow-up

(n =0)

Analyzed

(n=20)

**Analysis**

Analyzed

(n=20)

**Supplementary Material** Participants Flowchart.
